# Detailed Balance Limit of Efficiency of Broadband-Pumped Lasers

**DOI:** 10.1038/s41598-017-11857-y

**Published:** 2017-09-13

**Authors:** Sergey Nechayev, Carmel Rotschild

**Affiliations:** Department of Mechanical Engineering and Russell Berrie Nanotechnology Institute, Technion-Israel Institute of Technology, Haifa, 32000 Israel

## Abstract

Broadband light sources are a wide class of pumping schemes for lasers including LEDs, sunlight and flash lamps. Recently, efficient coupling of broadband light to high-quality micro-cavities has been demonstrated for on-chip applications and low-threshold solar-pumped lasers via cascade energy transfer. However, the conversion of incoherent to coherent light comes with an inherent price of reduced efficiency, which has yet to be assessed. In this paper, we derive the detailed balance limit of efficiency of broadband-pumped lasers and discuss how it is affected by the need to maintain a threshold population inversion and thermodynamically dictated minimal Stokes’ shift. We show that lasers’ slope efficiency is analogous to the nominal efficiency of solar cells, limited by thermalisation losses and additional unavoidable Stokes’ shift. The lasers’ power efficiency is analogous to the detailed balance limit of efficiency of solar cells, affected by the cavity mirrors and impedance matching factor, respectively. As an example we analyze the specific case of solar-pumped sensitized Nd^3+^:YAG-like lasers and define the conditions to reach their thermodynamic limit of efficiency. Our work establishes an upper theoretical limit for the efficiency of broadband-pumped lasers. Our general, yet flexible model also provides a way to incorporate other optical and thermodynamic losses and, hence, to estimate the efficiency of non-ideal broadband-pumped lasers.

## Introduction

Broadband free-space pumping is a conventional excitation scheme of optical resonators^1^, which recently gained momentum owing to the demonstration of effective broadband excitation of high-finesse on-chip micro-cavities^2^ and low-threshold solar-pumped lasers^3, 4^ (SPLs) via cascade energy transfer^5^. The optical efficiency of such lasers depends on multiple parameters^6–8^, including pump quantum efficiency, pump spatial and spectral overlap with the lasing mode and gain medium parameters. Nevertheless, the limiting efficiency of a particular pumping and gain medium configuration may be assessed from a purely thermodynamic point of view. In 1959^9^ the limiting efficiency of a 3-level maser in thermal contact with hot and cold thermal reservoirs was demonstrated to be that of a Carnot engine, confirmed by a more general treatment for lasers pumped by a heat reservoir^10^ and entropy balance of optically pumped lasers^11^. This approach, does not include the limited overall pump intensity available for the lasing system, neither does it include thermalisation losses for a broadband source. Both factors were elegantly encompassed by Shockley-Queisser^12^ derivation of the limiting efficiency of solar cells. Despite extensive research on the fundamental limit of broadband- pumped lasers (BPL)^13–19^, the effect of the thermodynamically dictated minimal Stokes’ shift^20^ on the maximal efficiency has not been explored, even though this consideration may have tremendous impact on the design of gain media.

In this paper we derive the detailed balance limit^12^ of efficiency for BPLs and show that lasers’ slope efficiency is analogous to the *nominal efficiency* in the Shockley-Queisser^12^ (SQ) derivation for semiconductors. Additionally, the laser optical-to-optical power efficiency, that takes into account the tradeoff between an output coupler reflectivity and a threshold population inversion, is analogous to the maximal power point of solar cell efficiency under an impedance matched load, hence the detailed balance limit of efficiency. We describe how the thermodynamic limit on BPLs slope efficiency is influenced by the absorption properties of the gain medium, pumping intensity, temperature and the thermodynamically unavoidable Stokes’ shift. Moreover we state that the conditions required for overall optical-to-optical efficiency to reach its thermodynamic limit are either infinitely high pump intensities, or that the ratio of the absorption constants at pump wavelength and lasing wavelength is infinite. We show explicitly how the ratio of the absorption constants influences the overall efficiency and discuss how to reduce these limitations via geometry. As an example of this formalism, we examine planar SPLs that operate under a variety of solar concentrations and solar-pumped Nd^3+^:YAG sensitized lasers^3^ and provide a way to incorporate optical and thermodynamic losses in our model. We conclude by comparison of our model to the state-of-the-art experimental results obtained by other groups.

**Figure 1:**
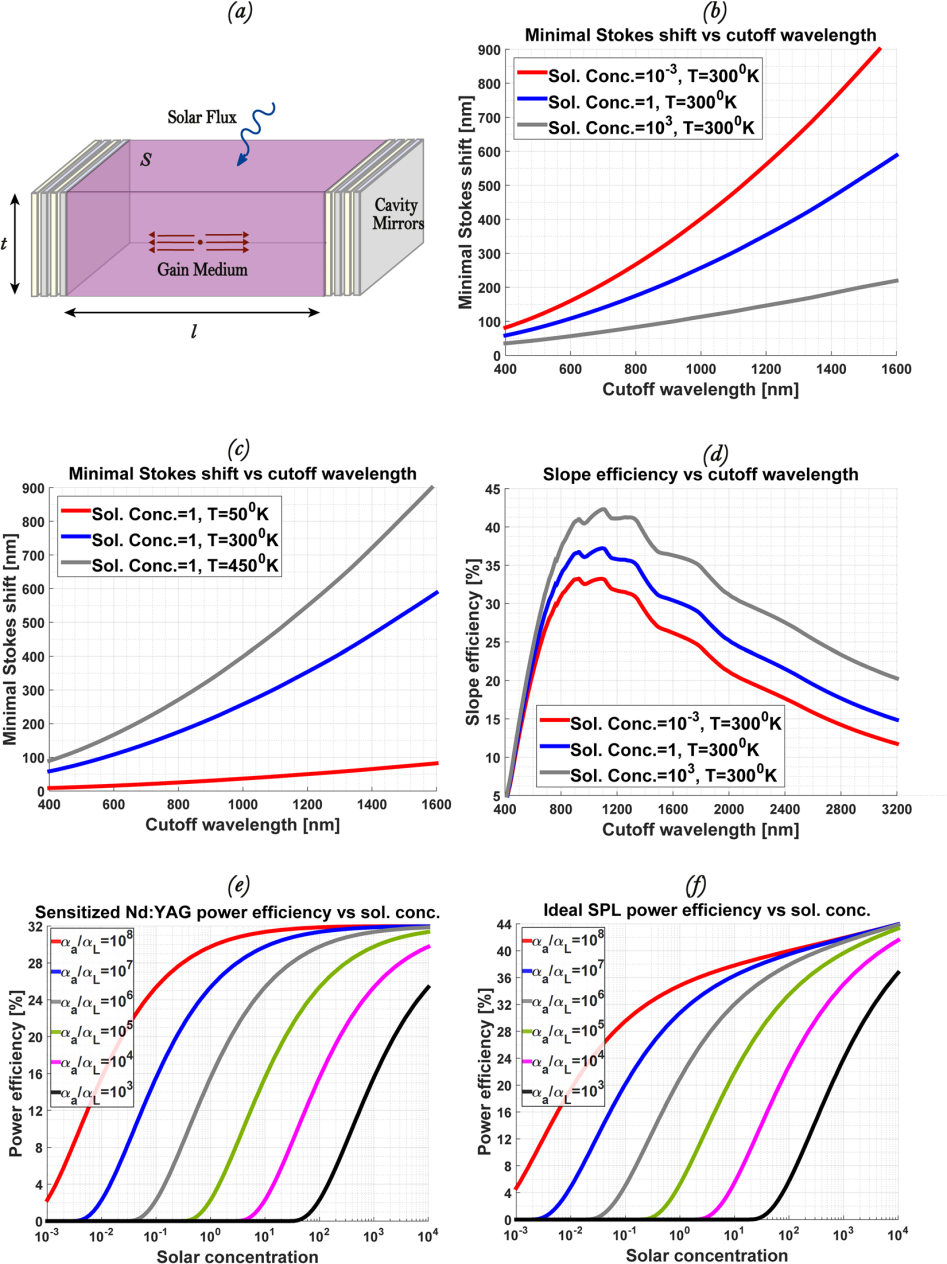
(**a**) A concept device: The pump light is absorbed by the gain medium pumped from the top. (**b**,**c**) Minimal Stokes’ shift between the absorption band edge and emission wavelength in solar-pumped lasers for different solar concentrations and temperatures as a function of the absorption band edge. (**d**) The thermodynamic limit of the SPLs slope efficiency for different solar concentrations at room temperature as a function of the absorption band edge. The peak value for unfocused solar illumination is 37% and it presents the detailed balance limit of efficiency for SPLs. (**e**,**f**) The resulting power efficiency of sensitized Nd^3+^:YAG laser and ideal SPL as a function of solar concentration for a variety of absorption constant ratios at room temperature.

The thermodynamic theory for BPLs was established in 1983, by Roxio and Yablonovitch^20, 21^, in the context of SPLs that operate under non-concentrated sunlight. The energy conservation law, Kirchhoff’s law of radiation^22–25^ and the principle of detailed balance^12^ for excited luminescent medium imply a constrain on such lasers’ emission wavelength. At non-zero temperature the lasers’ gain medium emission wavelength must be displaced from the absorption band edge by a Stokes’ shift that is larger than the thermodynamic minimum^20^, which is on a scale of hundreds of nanometers at room-temperature. The minimal Stokes’ shift is calculated from the solution of the integral inequality, stating that the gain medium luminescent emission rate per unit volume *L* (Eq. 1 LHS) should be less than the overall pump rate per unit volume^20^ *R* (Eq. 1 RHS):

1 $$\begin{array}{c}L={\int }_{\,}^{{\lambda }_{a}}[\alpha (\lambda ,\mu ,T)\frac{8\pi {n}^{2}}{{\lambda }^{2}}{\{\exp (\frac{hc/\lambda -\mu }{{k}_{B}T})-1\}}^{-1}]\frac{c}{{\lambda }^{2}}d\lambda \\ \le \frac{1}{V}{\int }_{\,}^{{\lambda }_{a}}[S\{1-\exp (-\alpha (\lambda ,\mu ,T)t)\}{\eta }_{p}(\lambda ,\mu ,T){I}_{P}(\lambda )]d\lambda =R\end{array}$$

where *h*, *c*, *k*_*B*_, *T*, *n*, *μ*, *λ* are Plank’s constant, the speed of light in vacuum, Boltzmann’s constant, the temperature of the medium, its refractive index, its chemical potential and wavelength, respectively. For the configuration of a planar waveguide laser pumped from the top (Fig. 1a) the geometrical parameters *V*, *S*, *t*, *l* are the volume of the excited medium, pumped surface area, pump propagation length, which is also the waveguide thickness, and cavity length, respectively. The values *α*(*λ*,*μ,* *T*), *I*_*P*_(*λ*), *η*_*p*_(*λ*, *μ*, *T*) present the absorption constant, pump photon flux (in photons per unit area per wavelength per second), and overall radiative quantum efficiency, respectively. Simplification is achieved when the value *η*_*p*_ also accounts for the gain medium quantum efficiency^26^, energy transfer efficiency^3, 4^ and other efficiency reduction mechanisms^6–8^.It is assumed that pump absorption follows the Beer-Lambert Law. The integration in Eq. 1 is performed over the range of wavelengths encompassed by the absorption band up to the cutoff wavelength *λ*_*a*_. It is further assumed that the excitation is weak^27^ and absorption coefficient is unsaturated^28^ *α*(*λ*, *μ*, *T*) = *α*(*λ*, 0, *T*), there is no fluorescence quenching^29^ *η*_*p*_(*λ*, *μ*, *T*) = *η*_*p*_(*λ*, 0, *T*) and that the pump is at threshold condition, i.e., *μ* = *hc*/*λ*_*L*_, where *λ*_*L*_ is the lasing wavelength. By solving the equation:

2 $$L({\lambda }_{L})-R=0$$

one obtains the minimal possible lasing wavelength *λ*_*L*_(*T*, *η*_*p*_, *λ*_*a*_, *n*, *I*_*P*_, *t*).

For an idealized device shown schematically in Fig. 1**a** pumped by a *C* − *times* concentrated pump source (*CI*_*p*_) with a constant absorption coefficient *α*(*λ*, *μ*, *T*) = *α*_*a*_ up to the absorption band cutoff wavelength *λ*_*a*_ and negligible afterwards, the inequality Eq. 1 can be integrated to give:

3 $${L}_{a}=\exp (\frac{hc/{\lambda }_{L}-hc/{\lambda }_{a}}{{k}_{B}T})\frac{8\pi {n}^{2}}{{{\lambda }_{a}}^{2}}(\frac{{k}_{B}T}{h})\le \frac{1-\exp (-{\alpha }_{a}t)}{{\alpha }_{a}t}{\int }_{\,}^{{\lambda }_{a}}[{\eta }_{p}C{I}_{p}(\lambda )]d\lambda ={R}_{a}$$

Hence, the minimal Stokes’ shift is

4 $${\rm{\Delta }}{\lambda }_{S}(T,\,{\eta }_{p},\,{\lambda }_{a},n,\,C{I}_{P},t)\equiv {\lambda }_{L}-{\lambda }_{a}={\lambda }_{a}^{2}\frac{{k}_{B}T}{hc}ln[\frac{8\pi {n}^{2}{k}_{B}T}{{\eta }_{p}{R}_{a}{{\lambda }_{a}}^{2}h}]$$

Notably Δ*λ*_*S*_ logarithmically increases with the reduction of either pump concentration *C* or quantum efficiency *η*_*p*_. Figure 1b and 1c show Δ*λ*_*S*_ as a function of absorption cutoff wavelength *λ*_*a*_ for a variety of pump concentrations and temperatures, assuming *I*_*P*_(*λ*) to be the solar photon flux, *η*_*p*_ = 100%, *n* = 1.82 and optically thin gain medium (*α*_*a*_ *t* ≪ 1). Making the gain medium optically thick *α*_*a*_ *t* ≫ 1 increases the minimal Stokes’ shift, since it is equivalent to the reduction of pump concentration for a given mode volume. Figure 1b and 1c and Eqs 1 and 4 show that the minimal Stokes’ shift can be a major limitation on the power efficiency for a gain medium at high temperature pumped by low intensity broadband illumination.

## Slope Efficiency of Broadband-Pumped Lasers

In order to expand the theory and include the Stokes’ shift impact on efficiency, we first define the slope efficiency as:

5 $${\eta }_{slope}={\eta }_{oc}V\frac{(hc/{\lambda }_{L})}{{P}_{in}}R$$

where *η*_*oc*_is the fraction of the total power that is coupled out of the laser^6–8^ and *V* is the lasing mode volume that is assumed to be equal to the pumped volume. Since the incident power is given by:

6 $${P}_{in}=S{\int }^{}{I}_{P}(\lambda )\frac{hc}{\lambda }d\lambda $$

where the integration encompasses all wavelengths, the slope efficiency can be rewritten in an intuitive form of:

7 $${\eta }_{slope}={\eta }_{oc}\frac{\frac{hc}{{\lambda }_{L}}{\int }_{\,}^{{\lambda }_{a}}[\{1-\exp (-\alpha (\lambda ,\mu ,T)t)\}\,{\eta }_{p}{I}_{P}(\lambda )]d\lambda }{{\int }^{}{I}_{P}(\lambda )\frac{hc}{\lambda }d\lambda }$$

Here the denominator represents the total pump power density incident on the resonator, while the nominator is the absorbed photon flux rate multiplied by the energy of the lasing photon. The reduced energy of the lasing photon, relative to the absorption band edge, accounts therefore not only for the thermalisation losses, but also for the unavoidable Stokes' shift obtained from Eq. 2. We find that the resulting slope efficiency *η*_*slope*_ is affected by the device temperature and pump concentration exclusively indirectly via the Stokes’ shift (Eq. 7). Notably, the slope efficiency *η*_*slope*_ at *η*_*oc*_ = 100% is the direct analogue of the *nominal efficiency*, i.e., the product of the open-circuit voltage and short-circuit current of a solar cell in the SQ derivation^12^. The additional Stokes’ shift reduces the laser’s slope efficiency in a similar way as the *ultimate* efficiency reduces to the *nominal* efficiency in SQ by the ratio of the open-circuit voltage to the ultimate voltage^12^ due to non-zero temperature, implying analogy. Figure 1d shows the resulting optimum slope efficiency *η*_*slope*_, maximized with respect to the gain medium thickness *t* using Eqs 4 and 7, for a variety of solar concentrations at *T* = 300 *K*, *η*_*p*_ = 100%, and negligible distributed cavity losses^6–8^ *η*_*oc*_ ~ 100%. The blue line in Fig. 1d shows the maximal value of the slope efficiency for unfocused illumination to be ~37% at room temperature. Notably, the optimal absorption cutoff wavelength *λ*_*a*_ ~ 1110 *nm* seen at Fig. 1d is almost independent of the temperature (not shown) or pump concentration for the specific case of solar illumination.

## Detailed Balance Limit of Efficiency of Broadband-Pumped Lasers

The laser’s optical-to-optical power efficiency *η*_*power*_ must include the loss associated with the need to maintain the cavity population inversion *N*_*t*_, hence

8 $${\eta }_{power}={\eta }_{oc}V\frac{(hc/{\lambda }_{L})}{({P}_{in})}[R-{N}_{t}({\eta }_{oc})/{\tau }_{2}]$$

where *τ*_2_ is the inverse of the decay rate per atom, ideally equal to the gain medium spontaneous emission lifetime *τ*_*sp*_. *N*_*t*_ is the population inversion of the cavity at threshold, given by *N*_*t*_ = *α*/*σ*, where *σ* is the gain medium emission cross-section at the lasing wavelength and $\alpha ={\alpha }_{L}-\frac{1}{2l}\,\mathrm{ln}(1-{t}_{oc})$ is the total resonator loss constant. Here *α*_*L*_ is the distributed loss constant at the lasing wavelength and *t*_*oc*_ is the output coupler transmittance. The quantity $\,{\eta }_{oc}=\frac{{t}_{oc}}{{L}_{i}+{t}_{oc}}$ presents the useful output of the optical resonator, i.e. the fraction of the total generated power in the cavity that is coupled out of the laser, where *L*_*i*_ ~ 2*α*_*L*_ *l* is the residual resonator’s loss^6–8^ per path. The useful output *η*_*oc*_ is close to unity for *t*_*oc*_≫  *L*_*i*_, however, rising *t*_*oc*_ eventually increases the power threshold of the resonator. Consequently, a practical system with a given pump source must be optimized for *η*_*power*_ through *t*_*oc*_ and the thickness of the gain medium *t*, which influences the tradeoff between the pump absorption and the mode volume of the resonator. In Eq. 8 both *η*_*power*_, *η*_*oc*_ are relevant only above the lasing threshold, i.e., if the condition *R> N*_*t*_ */τ*_2_ is satisfied. In this sense, the ratio of the power efficiency *η*_*power*_ to the *nominal efficiency*, i.e., the slope efficiency *η*_*slope*_ at *η*_*oc*_ = 100%:

9 $$\frac{{\eta }_{power}}{{\eta }_{slope}/{\eta }_{oc}}={\eta }_{oc}[1-\frac{{N}_{t}({\eta }_{oc})}{R{\tau }_{2}}]\le 1$$

is analogous to SQ impedance matching factor^12^, i.e., the ratio of the power output of a solar cell at maximal power point to the product of open-circuit voltage and short-circuit current, also known as *fill factor*. The ratio in Eq. 9 is strongly influenced by the gain medium and cavity parameters and can be close to unity for the case of a highly absorptive very low-threshold resonator^2^.

## Sensitized Nd^3+^:YAG-Like SPL and Ideal SPL

To demonstrate the presented model we consider a practical system resembling Nd^3+^:YAG SPL. The main absorption line of Nd^3+^:YAG is at 808 nm^30^, hence we assume that our sensitized Nd^3+^:YAG has an absorption band between 280 nm to 808 nm with absorption constant *α*_*a*_. Consequently, the Stokes’ shift of the sensitized Nd^3+^:YAG lasing at *λ*_*L*_ = 1064 *nm* ^26, 30–32^ is 256 nm, which is approximately *x*1.5 times larger than the minimally thermodynamically allowed value at non-concentrated solar illumination at room temperature. The rest of parameters are *η*_*p*_~100%, the loss constant at lasing wavelength $\,{\alpha }_{L}=3\cdot {10}^{-3}c{m}^{-1}$, the fluorescence lifetime *τ*_*sp*_ = 230 *μsec*, the length of the resonator *l* = 1*cm* and the emission cross-section at the lasing wavelength $\sigma =8.8\cdot {10}^{-19}c{m}^{2}$. Figure 1e shows the resulting power efficiency as a function of the pump flux density in units of solar concentration for optimal output coupler reflectivity *t*_*oc*_ and cavity thickness *t*. Maximal power efficiency achieved in Fig. 1e is ~31% and it may be reached under unfocused solar illumination only in the limiting case of ${\alpha }_{a}/{\alpha }_{L}\to \infty$. The slight reduction relative to the maximal slope efficiency value of ~37% shown in Fig. 1d is due to non-optimal cutoff wavelength and larger than minimal Stokes’ shift (Fig. 1b and 1c).

To present an ideal SPL we chose an optimal cutoff wavelength *λ*_*a*_ = 1110 *nm* (corresponding to the maximal values in Fig. 1d), where the lasing wavelength is displaced from the absorption cutoff wavelength *λ*_*a*_by the Stokes’ shift Δλ_*S*_ (Eq. 4 at *λ*_*a*_ = 1110 *nm*), hence *λ*_*L*_ = *λ*_*a*_ + Δλ_S_ and *σ*, *l*, *τ*_*sp*_, *η*_*p*_ are the same as for Nd^3+^:YAG. The power efficiency is the maximal value of the optical power output (Eq. 8) at given solar concentration in the parameters space that include the output coupler reflectivity *t*_*oc*_, cavity thickness *t* and the minimal Stokes’ shift Δλ_*S*_, that changes accordingly. The results are plotted in Fig. 1f at room temperature *T* = 300*K*. Notably, only in the limit of ${\alpha }_{a}/{\alpha }_{L}\to \infty$ the power efficiency *η*_*power*_ reaches its maximal value equal to the slope efficiency *η*_*slope*_. Therefore the maximal value of the slope efficiency of an ideal SPL under unfocused illumination *η*_*slope*,*max*_ = 37% (the maximal value of the blue line in Fig. 1d) presents the detailed balance limit of efficiency for SPLs operating under unconcentrated sunlight.

## Discussion and Conclusion

State-of-the-art SPLs operate far below the thermodynamic limit presented in the manuscript^33–35^. The slope efficiency is limited mostly by the thermalisation losses (Eq. 7) and therefore can reach almost the theoretical value by increasing the mode volume and the pump propagation distance in favor of full pump absorption (LASCAD simulations show^36^ *η*_*slope*_~30% for solar pumped Nd^3+^:YAG). However the power efficiency of state-of-the-art SPLs is almost an order of magnitude lower^33–35^. As it can be seen from Fig. 1e and 1f one of the most important parameters for optimal power output is the ratio of absorption constant at absorption band to that at lasing wavelength. This is due to the fact that lasers, especially BPLs, are subject to two competitive processes: the absorption of the pump in the gain media and the losses at the lasing wavelength, which scale simultaneously with the mode volume. Previously, it was shown^20^ that in order to allow lasing under non-concentrated solar illumination this ratio must be at least ${\alpha }_{a}/{\alpha }_{L} > {10}^{5}$. For lower ratios, all of the absorbed pump will be lost to maintain the threshold population inversion. Even if this criterion is satisfied, (Fig. 1e and 1f), for finite ratios *η*_*oc*_ must be chosen low enough to operate above threshold, impairing *η*_*power*_. Consequently, in order to achieve the limiting efficiency one must find a way to increase pump absorption without affecting the transparency at the lasing wavelengths. However, reaching the limit of ${\alpha }_{a}/{\alpha }_{L}\to \infty$, while maintaining limited Stokes’ shift may require an intelligent design of new materials and their energy transfer^2, 37^. For instance, in bandgap materials the absorption coefficient, *α*, below bandgap energy follows an exponential rule $\alpha ={\alpha }_{0}\exp (\frac{hc/\lambda -{E}_{a}}{{E}_{U}})$, where *α*_0_, *E*_*a*_ are material constants and *E*_*U*_ is the Urbach tail width^38–40^ that scales as *E*_*U*_ ∝ *k*_*B*_ *T*. Hence in this particular case the tradeoff must be considered between the absorption constants ratio ${\alpha }_{a}/{\alpha }_{L}$ and the Stokes’ shift, since requiring ${\alpha }_{a}/{\alpha }_{L}\to \infty$ at non-zero temperature leads to zero total efficiency due to infinite Stokes’ shift, while for the minimal Stokes’ shift the ratio ${\alpha }_{a}/{\alpha }_{L}$ is too low resulting in very high power threshold^41^.

By carefully examining the equations in the current manuscript and in ref. 20 one can see that for the special case of lasers pumped via cascade energy transfer^2–4^, or when the lasing mode volume is geometrically separated from the pump absorption region^17–19^, the absorption constants criterion must include the ratio of the absorption volume *V*_*a*_ to the lasing mode volume *V*_*L*_. Hence, the critical parameter for power efficiency would be $(\frac{{V}_{a}}{{V}_{L}})\cdot (\frac{{\alpha }_{a}}{{\alpha }_{L}})$ paving the way to overcome this constrain in practical conditions via intelligent design of a sensitizer^37^.

On the other hand, estimation of practical lasers’ efficiency takes into account factors that impair the energy transfer process^4, 6–8^. Apart from the unavoidable thermodynamic losses described above, our method allows incorporation of these efficiency reduction mechanisms via the “product of efficiencies” approach, assuming mutual independency of the efficiency reduction factors. First, the right-hand side of Eq. 1 must be integrated without the approximation of a thin cavity, taking into account the cavity thickness *t* and the wavelength-dependent absorption constant *α*(*λ*, *μ*, *T*). Second, we must account for the factor *η*_*p*_(*λ*) in the right-hand side of Eq. 1, which typically consists of a product of the pump quantum efficiency, energy transfer efficiency, waveguiding efficiency, etc. and was discussed in details in ref. 4. These limitations increase the minimal Stokes’ shift Δ*λ*_*S*_ derived from Eq. 2, however in practical system Δ*λ*_*S*_ can be even larger, since it is typically dictated by the gain medium properties. The slope efficiency *η*_*slope*_ in Eq. 5 must include the mode volume of the laser resonator instead of the volume of the bulk *V*, while the pump rate *R* in Eq. 5 will be substituted by the effective pump rate, affected by the same factors as *η*_*p*_(*λ*) and by the residual pumping to the lower laser level (see paragraph 6.4 of ref. 6 and refs 4, 7, 8). In the presence of non-radiative losses, the upper laser level is depleted faster than the rate of spontaneous emission (*τ*_2_ < *τ*_*sp*_), which increases the power threshold of the resonator, affecting Eqs 8 and 9. The impaired gain medium quantum efficiency (see paragraph IX in ref. 6) also reduces the effective pump rate, and clearly all subsequent efficiencies, via the reduction of *η*_*p*_. The equations in the manuscript may also include photon splitting^42^ (*η*_*p*_ > 1), temperature dependency^43^ or may be modified for other lasing configurations^6–8^.

In conclusion, we have presented a thermodynamic approach to estimate the efficiency of broadband-pumped lasers via the detailed balance limit approach. We examined the specific case of solar-pumped Nd^3+^:YAG-like sensitized lasers and shown limiting power efficiency of ~31%. We discussed the factors affecting the efficiency of broadband-pumped lasers and provided a way to incorporate them in the efficiency derivation. We have shown how the thermodynamically dictated Stokes’ shift affects the maximal slope efficiency, and how this Stokes’ shift and the ratio of absorption constants dictate the power efficiency of the broadband-pumped lasers. Specifically, we found that the maximal theoretical detailed balance limit of efficiency of SPLs is ~37% for unfocused illumination. The presented approach is generic and can be used to set the upper limit of efficiency for a specific combination of materials that constitute a laser with an arbitrary pump source. We foresee that our analysis may be useful for estimation of efficiency of other BPLs, such as led-pumped on-chip micro-lasers.

### Data availability statement

The authors declare that the data that support the findings of this study are available from the corresponding author upon reasonable request.
